# A New Trigonometric Spline Approach to Numerical Solution of Generalized Nonlinear Klien-Gordon Equation

**DOI:** 10.1371/journal.pone.0095774

**Published:** 2014-05-05

**Authors:** Shazalina Mat Zin, Muhammad Abbas, Ahmad Abd Majid, Ahmad Izani Md Ismail

**Affiliations:** 1 School of Mathematical Sciences, Universiti Sains Malaysia, Pulau Pinang, Malaysia; 2 Institute for Engineering Mathematics, Universiti Malaysia Perlis, Perlis, Malaysia; 3 Department of Mathematics, University of Sargodha, Sargodha, Pakistan; Georgia State University, United States of America

## Abstract

The generalized nonlinear Klien-Gordon equation plays an important role in quantum mechanics. In this paper, a new three-time level implicit approach based on cubic trigonometric B-spline is presented for the approximate solution of this equation with Dirichlet boundary conditions. The usual finite difference approach is used to discretize the time derivative while cubic trigonometric B-spline is applied as an interpolating function in the space dimension. Several examples are discussed to exhibit the feasibility and capability of the approach. The absolute errors and 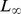
error norms are also computed at different times to assess the performance of the proposed approach and the results were found to be in good agreement with known solutions and with existing schemes in literature.

## Introduction

The generalized nonlinear Klien-Gordon (KG) equation arises in various problems in science and engineering. This paper focuses on the analysis and numerical solution of the generalized nonlinear KG equation, which is given in the following form [14]:
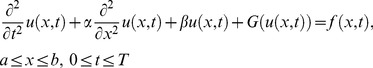
(1)subject to initial conditions
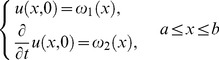
(2)and with Dirichlet boundary conditions
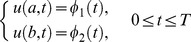
(3)where 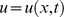 denotes the wave displacement at position and time 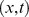, 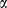 and 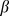 are real constants, 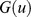 is a nonlinear function of 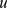 and 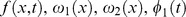 and 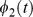 are known functions.

In particular, the KG equation is important in mathematical physics especially in quantum mechanics and it is well known as a soliton equation. A study on the interaction of soliton in collisionless plasma, the recurrence of initial states and examination of the nonlinear wave equations was in [1].

Several methods, in addition to several finite difference schemes, have been developed to solve the nonlinear KG equation. Jiminez and Vazquez [2] introduced four numerical schemes for solving the nonlinear KG equation. Ming and Guo utilized a Fourier collocation method for solving the nonlinear KG equation [3]. The KG equation was approximated using decomposition scheme by Deeba and Khuri [4] and using the Legendre spectral method by Guo et al [5]. Wong et. al. solved an initial value problem involving the nonlinear KG equation using fully implicit and discrete energy conserving finite difference scheme [6]. Wazwaz introduced the tanh and sine-cosine method to obtain compact and noncompact solutions for the nonlinear KG equation [7]. Sirendaoreji solved the nonlinear KG equation using the auxiliary equation method to construct new exact traveling wave solutions with quadratic and cubic nonlinerity [8]. Yucel solved the nonlinear KG equation using homotopy analysis method [9] and Chowdhury and Hashim solved the equation using homotopy-pertubation method [10].

B-spline functions can be used to solve numerically linear and non-linear differential equations. Caglar et. al. [11] has introduced a cubic B-spline interpolation method to solve the two-point boundary value problem. Hamid et al. [12] has introduced an alternative cubic trigonometric B-spline interpolation method to solve the same problem. Dehghan and Shokri [13] have obtained a numerical solution of the nonlinear KG equation using Thin Plate Splines radial basis functions. Khuri and Sayfy [14] have solved the generalized nonlinear KG equation using a finite element collocation approach based on third degree B-spline polynomials.

In this work, a new three-time level implicit approach which combines a finite difference approach and cubic trigonometric B-spline collocation method (CTBCM) is proposed to solve generalized nonlinear KG equation. The finite difference approach is proposed to discretize time derivative and cubic trigonometric collocation method is applied to interpolate the solutions at time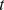. Two numerical experiments are carried out to calculate the numerical solutions, absolute errors, 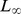 error norms and order of convergence for each problem in order to show the accuracy of method.

### Temporal Discretization

Consider a uniform mesh 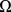 with grid points 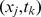 to discretize the grid region 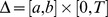 with 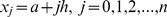 and 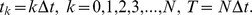 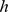 and 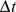 denote mesh space size and time step size respectively. The time derivative is approximated using the central finite difference formula
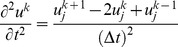
(4)


Using the approximation of equation (4), equation (1) becomes
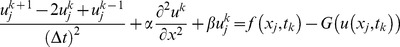
(5)


Using the 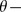
weighted technique, the space derivatives of equation (5) becomes
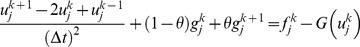
(6)where 
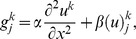
 
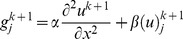
, 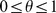 and the subscripts 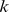 and 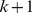 are successive time levels.

After simplification, equation (6) leads to
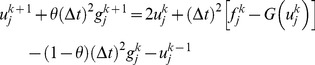
(7)


### Trigonometric B-Spline Collocation method

In this section, CTBCM is used to solve Klien-Gordon equation. Cubic trigonometric B-spline are used to approximate the space derivatives. To construct the numerical solution, nodal points 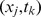 defined in the region 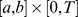 where 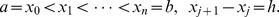


The approximate solution 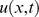 to the exact solution 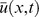 is defined as [18]:
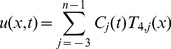
(8)where 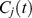 are time dependent unknowns to be determined and 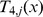 are cubic trigonometric B-spline basis function given as:
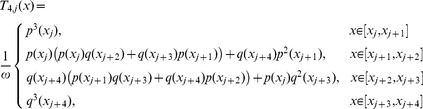
(9)where
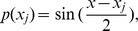


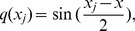


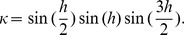



Due to local support properties of B-spline basis function, there are only three non-zero basis functions 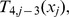 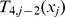 and 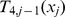 are included over subinterval 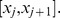 Thus, the approximation 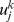 and its derivatives with respect to *x* can be simplified as:
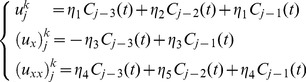
(10)where
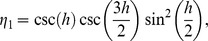


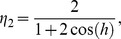


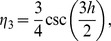


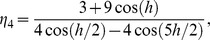


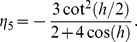



The solution to problem (1) is obtained by substituting equation (10) into equation (7). Initially, time dependent unknowns 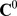 are calculated and shown in section 3.1. Next, the following initial condition is substituted into the last term of equation (7) for computing 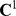


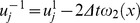
(10a)


Subsequently, the time dependent unknowns, 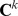 for 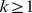 should be generated. After simplification, equation (7) leads to
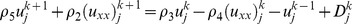
(11)where,
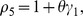


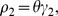


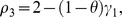


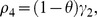


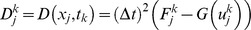
with
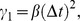


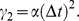



The system obtained on simplifying (11) consists of 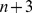 unknowns 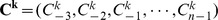 in 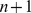 linear equations at the time level 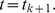 In order to obtain a unique solution, the equation (8) is applied to the boundary conditions given in equation (3) for two additional linear equations.
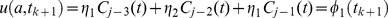
(12)

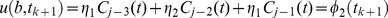
(13)


From equations (11), (12) and (13), the system can be written as
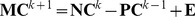
(14)where,
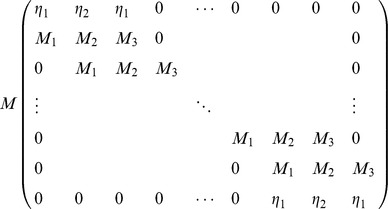


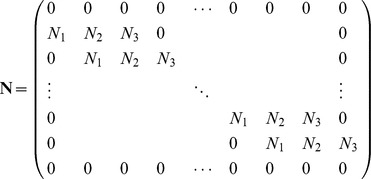


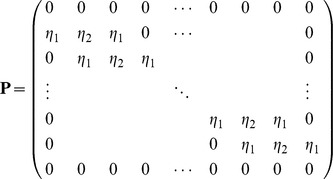


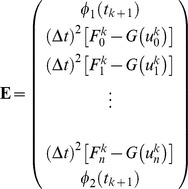



With
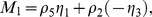


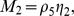


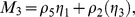


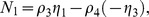


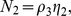


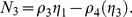



Half explicit and half implicit scheme is produced by choosing 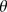 to be 0.5. This scheme is known as Crank-Nicolson scheme. System (14) becomes a tri-diagonal matrix system of dimension 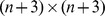 that can be solved using the Thomas Algorithm [17].

### Initial vector 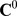


The initial vectors 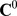
can be obtained from the initial condition as well as boundary values of the derivatives of the initial condition [11], [15]:
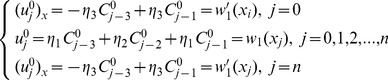
(15)


This yields a 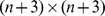
matrix system:
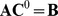


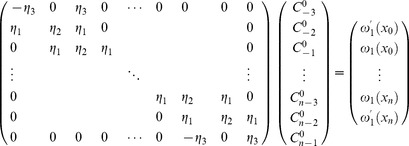
(16)


The solution can be obtained by using the Thomas Algorithm [17].

### Stability analysis using von Neumann method

The von Neumann analysis of stability considers the growth of error in a single Fourier mode [19]–[20]

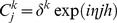
(17)where 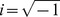 and 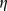 is the mode number. It is known that this method can be used to analyze the stability of linear scheme. All the nonlinear term in (11) are assumed to be zero [19]. Thus, equation (11) becomes
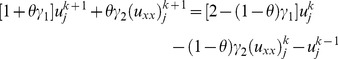
(18)where 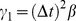 and 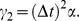 Substituting the approximate solution (8) in equation (18) leads to
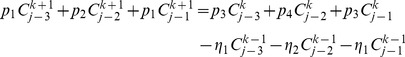
(19)where
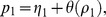


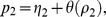


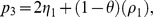


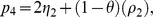


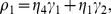


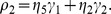



In order to obtain the amplification factor 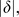 the trial solution (17) is substituted in (19) and after some simplification, we obtain,
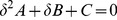
(20)where 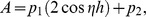 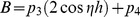 and 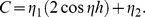 Since 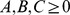 and 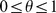, the amplification factor of this scheme is
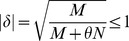
(21)where 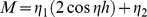 and 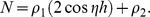 Hence, this scheme is unconditionally stable.

### Numerical Results and Discussions

In this section, the CTBCM is applied on two numerical problems. In order to measure the accuracy of the method, absolute errors and 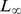
error norms are calculated using the following formulas [16]

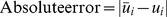
(22)

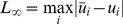
(23)where 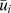 and 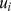 are analytical solution and approximate solution of proposed problem (1), respectively. The numerical order of convergence, *p* is obtained by using following formula [14]

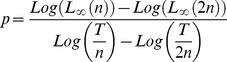
(24)where 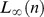 and 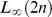 are the 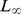 at number of partition *n* and *2n* respectively.

### Problem.1

Consider the following nonlinear Klien-Gordon equation [13], [14]

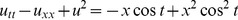
(25)subject to the following boundary and initial conditions
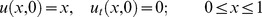


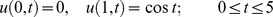



**Figure 1:**
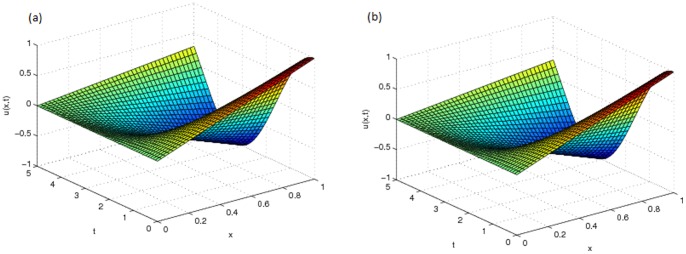
(a) Analytical solution, 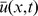 of Problem 1 (b) Approximate solution, 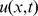 of Problem 1 with 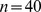 and 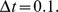.

**Figure 2:**
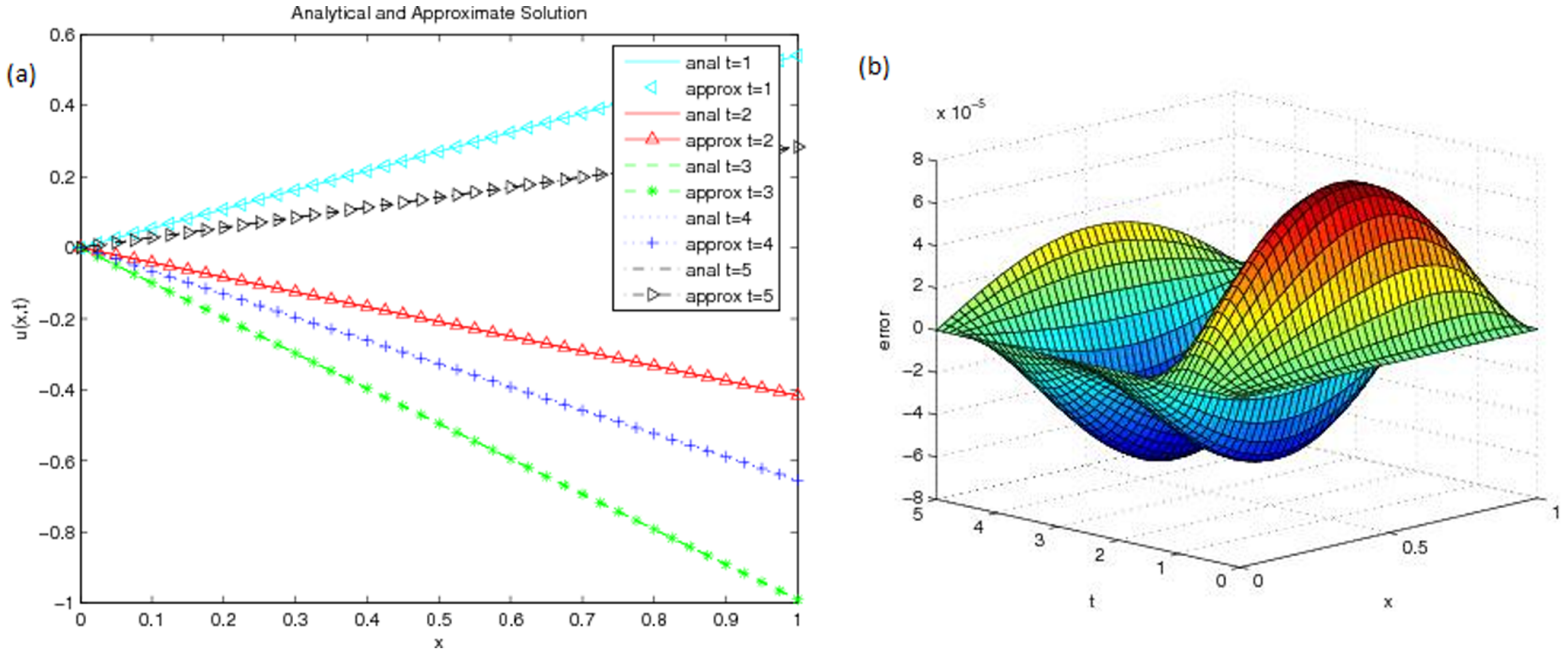
(a) Analytical and approximate solution at different time (b) Error of Problem 1 with 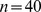 and 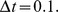.

The analytical solution of problem (25) is known to be 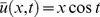 and graphically shown in Fig. 1 (a). This problem is tested using different values of 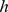 and 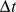 to show the capability of the present method. The final time is taken to be 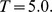 Fig. 1 (b) and Fig. 2 (a) show the approximate solution and Fig. 2 (b) shows the error of this problem with 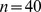 and 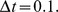 Two cases of this problem are discussed where Case 1 and Case 2 consider 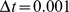 and 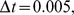
respectively. Numerical solutions, absolute errors and order of convergence of each case are tabulated in Tables 1–4 and Tables 5–8. The 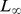
error norms are compared to Dehghan and Shokri [13] and Khuri and Sayfy [14] in Table 3 and Table 7. The comparison indicates that the present method is more accurate. The order of convergence of the present problem is calculated by the use of the formula given in (24) and is tabulated in Table 4 and Table 8. An examination of these tables indicates the method has a nearly second order of convergence.

**Table 1:** Numerical solution of Problem 1 at 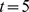
_._

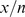	0	0.2	0.4	0.6	0.8	1.0
10	0	0.0567	0.1134	0.1702	0.2269	0.2837
20	0	0.0567	0.1135	0.1702	0.2269	0.2837
40	0	0.0567	0.1135	0.1702	0.2269	0.2837
80	0	0.0567	0.1135	0.1702	0.2269	0.2837
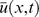	0	0.0567	0.1135	0.1702	0.2269	0.2837

**Table 2:** Absolute error of Problem 1 at 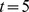
_._

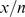	0	0.2	0.4	0.6	0.8	1.0
10	0	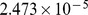	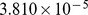	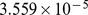	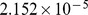	0
20	0	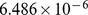	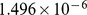	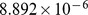	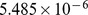	0
40	0	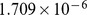	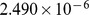	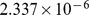	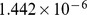	0
80	0	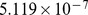	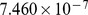	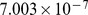	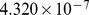	0

**Table 3:** Comparison of 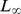 errors norms with Khuri & Sayfy [14] using 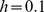, 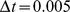 and Dehghan and Shokri [13] using 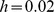, 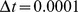
_._

t	1	2	3	4	5
Dehghan& Shokri [13]	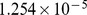	--------------	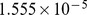	-------------	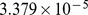
Khuri& Sayfy [14]	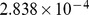	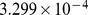	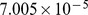	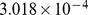	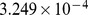
Present method	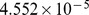	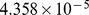	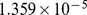	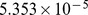	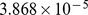

**Table 4:** The maximum 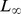 errors norms and order of convergence, *p* for Problem 1.

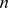	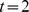		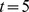	
	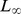	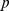	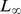	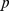
10	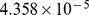	--------	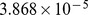	---------
20	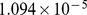	1.9942	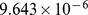	2.0039
40	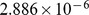	1.9927	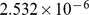	1.9290
80	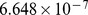	1.7384	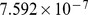	1.7379

**Table 5:** Numerical solution of Problem 1 at 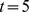
_._

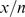	0	0.2	0.4	0.6	0.8	1.0
25	0	0.0567	0.1134	0.1702	0.2269	0.2837
50	0	0.0567	0.1135	0.1702	0.2269	0.2837
100	0	0.0567	0.1135	0.1702	0.2269	0.2837
200	0	0.0567	0.1135	0.1702	0.2269	0.2837
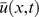	0	0.0567	0.1135	0.1702	0.2269	0.2837

**Table 6:** Absolute errors of Problem 1 at 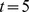
_._

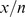	0	0.2	0.4	0.6	0.8	1.0
25	0	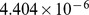	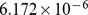	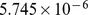	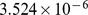	0
50	0	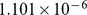	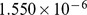	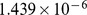	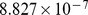	0
100	0	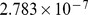	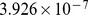	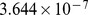	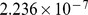	0
200	0	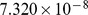	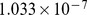	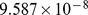	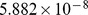	0

**Table 7:** Comparison of 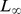 errors norms with Khuri & Sayfy [14] using 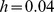, 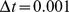 and Dehghan and Shokri [13] using 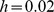, 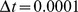
_._

t	1	2	3	4	5
Dehghan& Shokri [13]	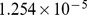	------------	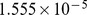	------------	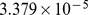
Khuri& Sayfy [14]	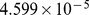	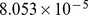	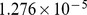	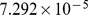	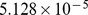
Present method	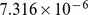	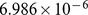	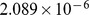	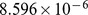	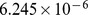

**Table 8:** The maximum 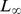 error norms and order of convergence, *p* for Problem 1.

n	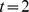		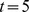	
	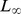	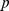	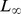	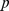
25	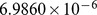	---------	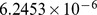	--------
50	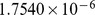	1.9938	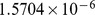	1.9916
100	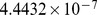	1.9810	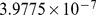	1.9812
200	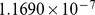	1.9263	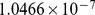	1.9262

#### Case.1

Numerical solutions, absolute errors, 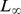
error norms and order of convergence using time step size 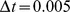


#### Case.2

Numerical solutions, absolute errors, 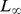 error norms and order of convergence using time step size 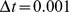


### Problem.2

The following nonlinear Klien-Gordon equation which is also known as Sine-Gordon equation is considered [14]

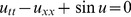
(26)


It is subjected to initial conditions and boundary conditions as
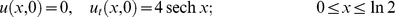


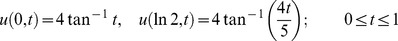



**Figure 3:**
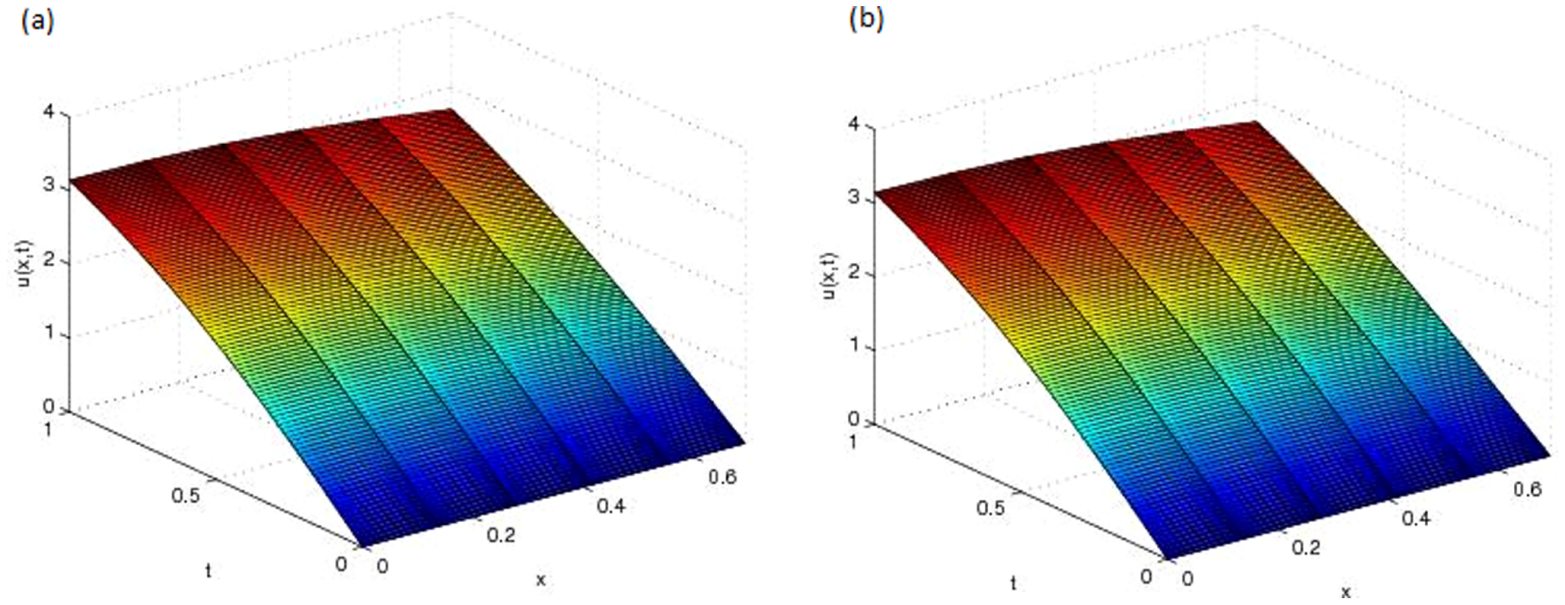
(a) Analytical solution, 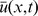 of Problem 2 (b) Approximate solution 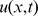 of Problem 2 with 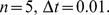.

**Figure 4:**
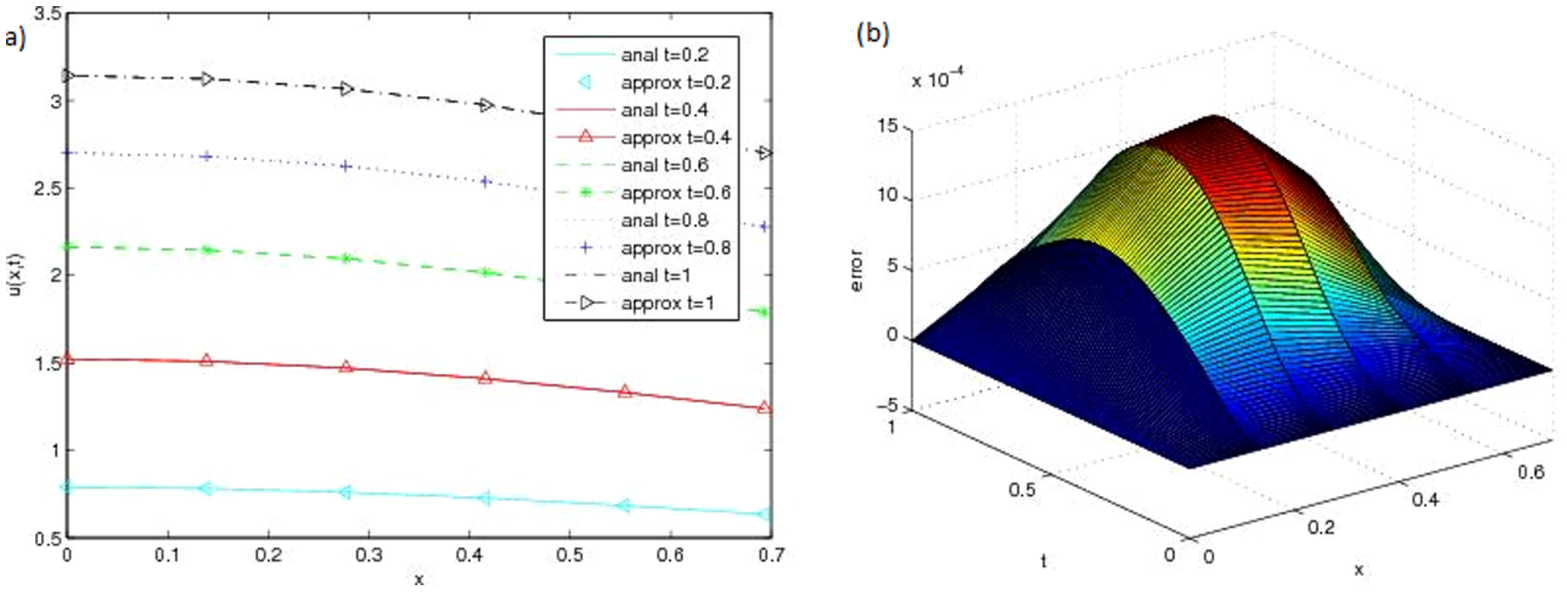
(a) Analytical and approximate solution at different time (b) Error of Problem 2 with 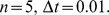.

The analytical solution of this problem is 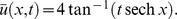 Fig. 3 (a) depicts a graph of this analytical solution. The final time is taken as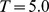. The approximate solutions are calculated at time step size 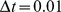
with different mesh space size, *h*. Numerical solutions are recorded in Table 9 and graphical solutions are plotted in Fig. 3 (b) and Fig. 4 (a). Absolute errors are calculated and shown in Table 10 while the 3D error plot is depicted in Fig. 4 (b). Table 11 shows the comparison of 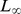 error norms between the present method with Khuri and Sayfy [14] method. This comparison shows that the present method gives better results.

**Table 9:** Numerical solution of Problem 2 at 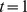
_._

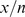	0	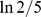	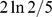	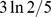	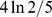	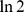
5	3.1416	3.1222	3.0654	2.9733	2.8495	2.6990
10	3.1416	3.1223	3.0655	2.9734	2.8496	2.6990
20	3.1416	3.1223	3.0655	2.9734	2.8496	2.6990
40	3.1416	3.1223	3.0655	2.9734	2.8496	2.6990
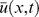	3.1416	3.1224	3.0657	2.9736	2.8497	2.6990

**Table 10:** Absolute error of Problem 2 at 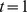
_._

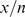	0	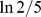	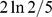	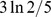	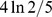	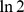
5	0	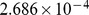	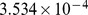	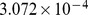	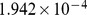	0
10	0	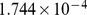	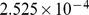	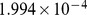	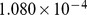	0
20	0	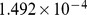	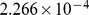	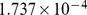	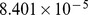	0
40	0	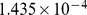	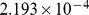	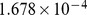	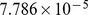	0

**Table 11:** Comparison of 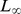 errors norms with Khuri and Sayfy [14] using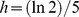, 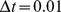
_._

t	0.01	0.02	0.10	0.50	1.00
Khuri& Sayfy [14]	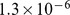	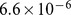	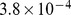	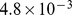	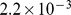
Present method	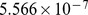	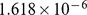	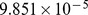	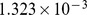	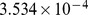

## Conclusions

In this work, Klien-Gordon equation has been successfully solved using CTBCM incorporating a finite difference scheme. Specifically, the central difference approach is used to discretize the time derivatives and cubic trigonometric B-spline is used to interpolate the solutions at displacement 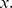 Well-known two test problem were solved using the proposed method and the solution obtained were in good agreement with the known solution. Accurate solutions at intermediate points can be easily obtained.
